# 907 Social Determinants Screening in an Outpatient Burn Setting: A Stopgap or a Durable Solution?

**DOI:** 10.1093/jbcr/iraf019.438

**Published:** 2025-04-01

**Authors:** Lauren Nosanov, James Walker, Jourden Martin, Afekwo Ukuku, Carey Lamphier, Yuk Ming Liu, Laura Johnson

**Affiliations:** Emory University School of Medicine; Emory University School of Medicine; Morehouse School of Medicine; Grady Health System; Grady Health System; Walter L. Ingram Burn Center at Grady Memorial Hospital; Walter L. Ingram Burn Center at Grady Memorial Hospital

## Abstract

**Introduction:**

Holistic care of Burn patients requires appreciation of the complex interplay between injury characteristics, patient-specific medical factors, systems issues, and socioeconomic and psychosocial considerations. Social determinants of health (SDoH) encompass non-medical factors that influence health outcomes and have been demonstrated to drive inequity in healthcare. Providing equitable and optimal care to burn patients requires proactive identification of SDoH.

**Methods:**

A system-wide SDoH screening process was introduced at a large urban safety-net hospital. Retrospective review was performed of prospectively collected data on all patients screened in our outpatient Burn Center clinic between July 2023-July 2024. Patients were categorized as either burn or non-burn (comprising skin sloughing diseases, soft tissue infections, road rash, and other etiologies). Cases were cross-referenced with patient and injury information present in the institutional Burn Center registry. Screening survey responses were compared for admitted patients and those managed completely outpatient. Univariate analysis and binary logistic regression identified independent predictors of positive screens.

**Results:**

Screening was performed on 1010 patients during the one-year study period, 37.8% of which had at any point previously been admitted to the inpatient Burn service. Median age was 42.0 years, with 64.1% being male; burn patients comprised 89.4% (median TBSA 5.5%). Financial resource strain was reported by 46.3%, 30.2% had experienced food scarcity in the prior year, and 25.6% had struggled with paying rent/mortgage. Approximately half of both admitted and non-admitted patient groups screened positive for at least one SDoH. Older age and insurance status independently predicted positive screens.

**Conclusions:**

Outpatient screening of patients cared for by our Burn Center identified high rates of SDoH and corresponding need for support. Given that patients seen in the Emergency Department and those cared for inpatient may not follow up in clinic, SDoH screening should be expanded to these settings. Next steps include exploration of unmet SDoH needs unique to the burn patient population not currently captured by hospital system-wide screening.

**Applicability of Research to Practice:**

Quality improvement efforts regarding clinical outcomes in Burn Center patients must proactively encompass SDoH screening and intervention to achieve holistic and optimal care.

**Funding for the Study:**

N/A

